# Real-word study of racial/ethnic disparities and socioeconomic determinants of overall survival in male breast cancer

**DOI:** 10.21203/rs.3.rs-5808248/v1

**Published:** 2025-02-21

**Authors:** Jincong Q. Freeman, Kent Schechter, Long C. Nguyen, Olasubomi J. Omoleye, Jared H. Hara

**Affiliations:** The University of Chicago; The University of Chicago; The University of Chicago; The University of Chicago Medical Center; The Queen’s Medical Center

## Abstract

This study assessed racial/ethnic disparities and socioeconomic determinants of overall survival in male breast cancer. Using the 2010–2021 US National Cancer Database, we identified 20,470 patients: 78.2% White, 13.8% Black, 4.0% Hispanic, and 2.5% Asian or Pacific Islander. After adjusting for clinicopathologic characteristics, Black patients had higher mortality than White patients (adjusted hazard ratio [AHR] 1.22, 95% CI: 1.12–1.32); however, when further adjusting for socioeconomic factors, this difference was no longer significant (AHR 1.09, 95% CI: 0.99–1.21). Hispanic patients had better survival. In the TNBC cohort, Asian or Pacific Islander patients had higher mortality than White patients (AHR 2.35, 95% CI: 1.21–4.55), warranting further investigation. In this US male breast cancer cohort, Black patients and White patients had similar mortality risk after further adjusting for socioeconomic indicators. Higher median household income and private insurance were linked to better survival. Strategies addressing socioeconomic inequities may help improve male breast cancer outcomes.

## Introduction

Male breast cancer (mBC) is rare, constituting less than 1% of all breast cancer cases in the United States (US) with an estimated 2,800 new cases in 2023.^1^ Men who identify as Black or African American have higher rates of breast cancer than those who identify as White, Hispanic, and Asian or Other Pacific Islander.^2^ Due to its rarity, data on mBC outcomes by race/ethnicity and socioeconomic determinants is limited. Additionally, due to comparatively limited evidence, mBC often gets treated following the guidelines created for female breast cancer. Hormone receptor (HR)-positive mBC – similar to estrogen receptor-positive, postmenopausal breast cancer in women – is treated with tamoxifen and/or chemotherapy. Some patients with triple-negative breast cancer (TNBC) may be eligible for pembrolizumab. mBC tumors with specific genetic mutations (e.g., human epidermal growth factor receptor 2 [HER2], PD-1, and *PIK3CA*) inform the use of targeted therapies or immune checkpoint inhibitors.^3^ Evidence suggests that, although similar in some regards, mBC differs in its biological and clinical behavior compared to female breast cancer,^4^ highlighting the need for more focused research to better understand its distinct characteristics and differences to improve male patients’ outcomes.

Although less common than female breast cancer, the incidence of mBC has risen in recent years,^5,6^ driven in part by known risk factors such as family history, aging, obesity, and high-penetrance genes (i.e., *BRCA1* and *BRCA2*) that can elevate the risk for mBC by up to 80-fold.^7^ Previous studies also have documented that mBC patients have lower 3-year and 5-year survival rates than female breast cancer patients for all stages of disease.^8–10^ Limited studies of racial/ethnic disparities in mBC mortality have indicated that Black men experience worse survival outcomes than White men,^11–13^ mirroring trends seen among women with breast cancer in the US.^14^

Social determinants have been shown to contribute to disparities in female breast cancer mortality, including socioeconomic status, access to health care and services, and facility type.^6^ While race and ethnicity are closely tied to socioeconomic factors in the US, addressing these factors reduces racial/ethnic disparities in female breast cancer risk and health outcomes.^15^ As previous studies in mBC have largely focused on differences between White patients and Black patients and the HR-positive/HER2-negative molecular subtype, little to less is known about mBC mortality disparities in other racial/ethnic groups and other molecular subtypes.^11,16,17^ To address these gaps, we examined disparities in overall survival (OS) of mBC by race/ethnicity and social determinants across tumor stages and molecular subtypes, using a large US clinical oncology registry.

## Results

### Patient characteristics

We identified a total of 20,470 mBC patients. The mean age at diagnosis was 66.2 years (SD 12.6). Most (78.2%) patients self-identified as White, followed by 13.8% as Black, 4.0% as Hispanic, 2.5% as API, and 1.6% as Other (Table 1). Overall, 40.2% were at a median household income quartile of ≥$63,333; 37.3% had private insurance, 52.3% were on Medicare while 4.9% were on Medicaid; 91.4% were diagnosed with stage I-III; 83.6% were HR-positive/HER2-negative; and 54.1% had grade 2 tumors (Table 1). Compared with White patients, API, Black, or Hispanic patients were diagnosed at younger age, at higher percent no high school degree quartiles, at lower median household income quartiles (except for API), were more likely to be uninsured or on Medicaid (Table 2). Black patients and Hispanic patients were more likely to be diagnosed with TNBC and grade 3 tumors compared to other racial/ethnic groups (Table 2).

### Racial/ethnic and socioeconomic disparities in mortality

**Fig. 1:**
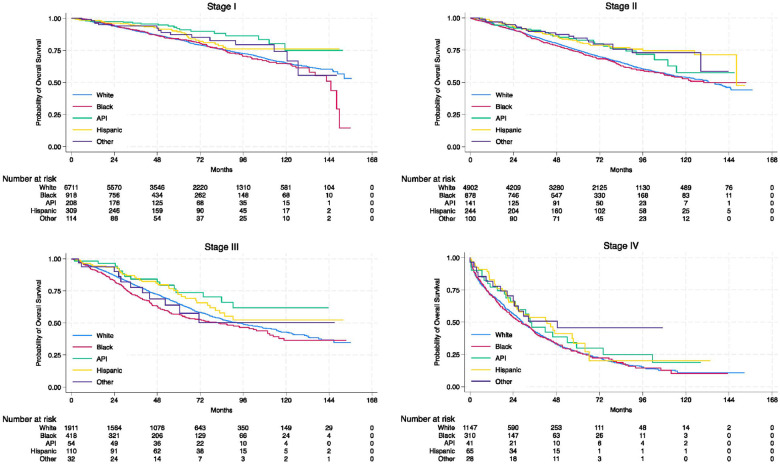
Kaplan-Meier curves for overall survival of male patients with breast cancer across racial/ethnic groups, stratified by tumor stage. Abbreviations: API, Asian or Pacific Islander; HR, hormone receptor; HER2, human epidermal growth factor receptor 2; TNBC, triple-negative breast cancer.

**Fig. 2:**
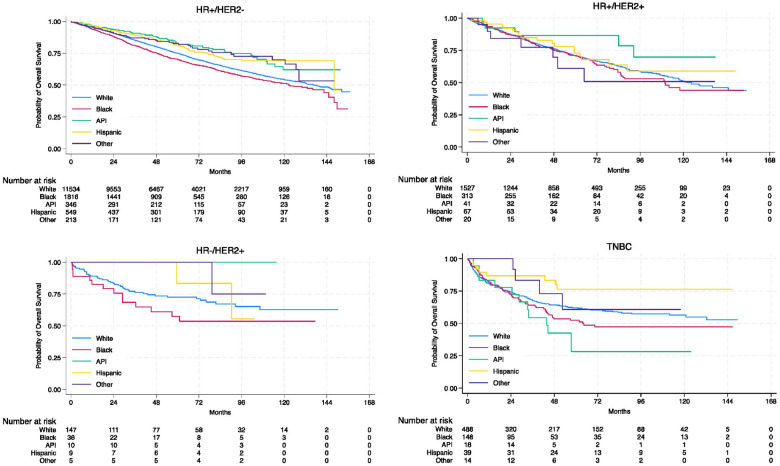
Kaplan-Meier curves for overall survival of male patients with breast cancer across racial/ethnic groups, stratified by tumor molecular subtype. Abbreviations: API, Asian or Pacific Islander; HR, hormone receptor; HER2, human epidermal growth factor receptor 2; TNBC, triple-negative breast cancer.

With a median follow-up of 52.8 months (IQR: 27.7–84.6), there were differences in OS between racial/ethnic groups overall (Supplementary Fig. 2), with Black patients having the shortest median survival (113.0 months [95% CI: 106.7–130.0]) (Supplementary Table 1). When stratified by tumor stage, Black patients experienced worse OS than other racial/ethnic patients in stage I, II, and III cohorts (Fig. 1); the OS rate was similar by race/ethnicity for stage IV disease (Supplementary Table 1). When stratified by molecular subtype, we observed OS differences across racial/ethnic groups in the HR-positive/HER2-negative and TNBC cohorts (Fig. 2). Black patients in the HR-positive/HER2-negative cohort and API patients in the TNBC cohort had the shortest median survival (Supplementary Table 1). Among all racial/ethnic groups, Black patients had the lowest 3-year, 5-year, and 10-year rates of OS overall; however, these rates vary across tumor stages and molecular subtypes (Supplementary Table 2).

Overall (Supplementary Table 3), mortality risk after adjusting for clinicopathologic characteristics (model 2) was higher in Black patients (AHR 1.22, 95% CI: 1.12–1.32, *P* < 0.001) and lower in API patients (AHR 0.69, 95% CI: 0.54–0.88, *P* = 0.003) compared to White patients. When further adjusting for socioeconomic factors (model 3), the OS difference was no longer significant between Black patients and White patients (AHR 1.09, 95% CI: 0.99–1.21, *P* = 0.075); API patients (AHR 0.70, 95% CI: 0.54–0.90, *P* = 0.006) and Hispanic patients (AHR 0.76, 95% CI: 0.62–0.94, *P* = 0.010) had a lower mortality risk (Supplementary Table 3). Compared to patients with a median household income of <$40,227, those of $40,227-$50,353 (AHR 0.89, 95% CI: 0.80–0.99, *P* = 0.038), $50,354-$63,332 (AHR 0.85, 95% CI: 0.76–0.96, *P* = 0.006), or ≥$63,333 (AHR 0.76, 95% CI: 0.67–0.86, *P* < 0.001) had a lower risk of mortality. Patients with no insurance (AHR 1.79, 95% CI: 1.42–2.26, *P* < 0.001), Medicaid (AHR 1.60, 95% CI: 1.35–1.89, *P* < 0.001), or Medicare (AHR 1.19, 95% CI: 1.09–1.30, *P* < 0.001) had a higher mortality risk than those privately insured. Greater comorbidity scores were associated with worse OS (Supplementary Table 3).

**Fig. 3:**
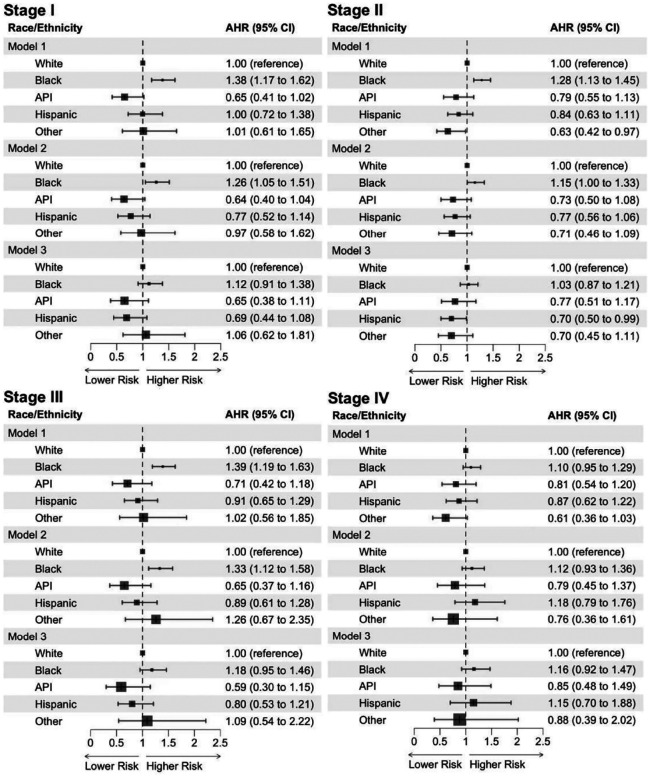
Racial/ethnic differences in overall survival of male patients with breast cancer, stratified by tumor stage: Cox proportional hazards regression. Abbreviations: API, Asian or Pacific Islander; AHR, adjusted hazard ratio; Cl, confidence interval. **Model 1** included age at diagnosis only. **Model 2** included age at diagnosis, histologic type, molecular subtype, tumor grade, and Charlson-Deyo comorbidity score. **Model 3** included age at diagnosis, histologic type, molecular subtype, tumor grade, Charlson-Deyo comorbidity score, percent no high school degree quartile, median household income quartile, type of health insurance, rural-urban area, and facility type.

When adjusting for clinicopathologic factors in Model 2, Black patients had a greater risk of mortality with stage I (AHR 1.26, 95% CI: 1.05–1.51, *P* = 0.011), stage II (AHR 1.15, 95% CI: 1.004–1.33, *P* = 0.044), and stage III tumors (AHR 1.33, 95% CI: 1.12–1.58, *P* = 0.001) compared to White patients (Fig. 3). However, after adjustment for socioeconomic factors in Model 3, this difference was no longer significant across stages I (AHR 1.12, 95% CI: 0.91–1.38, *P* = 0.281), II (AHR 1.03, 95% CI: 0.87–1.21, *P* = 0.758), and III (AHR 1.18, 95% CI: 0.95–1.46, *P* = 0.132) (Supplementary Tables 4–6). No significant difference in mortality was observed by race/ethnicity for patients with stage IV tumors (Supplementary Table 7). Compared to patients with a median household income of <$40227, patients with a median household income of ≥$63,333 and either stage I (AHR 0.73, 95% CI: 0.58–0.93, *P* = 0.012) or stage II tumors (AHR 0.72, 95% CI, 0.59–0.88, *P* = 0.001) had lower mortality risks (Supplementary Tables 4 and 5). For stages III and IV, no significant differences in mortality between median income quartiles were observed (Supplementary Tables 6 and 7).

In patients with stage I tumors, Medicaid was associated with a greater mortality risk compared to private insurance (AHR 1.72, 95% CI: 1.16–2.57, *P* = 0.007) (Supplementary Table 4). With stage II tumors, patients uninsured (AHR 1.97, 95% CI: 1.34–2.91, *P* = 0.001) or on Medicare (AHR 1.18, 95% CI: 1.02–1.36, *P* = 0.028) had a higher risk of mortality compared to private insurance (Supplementary Table 5). For stage III disease, Medicaid was associated with a greater mortality risk compared to private insurance (AHR 1.52, 95% CI: 1.08–2.15, *P* = 0.016) (Supplementary Table 6). In stage IV disease, lack of insurance, Medicare, or Medicaid was associated with increased mortality risk (Supplementary Table 7). Patients with a Charlson-Deyo comorbidity score of >2 consistently had a greater mortality risk across all tumor stages (Supplementary Tables 4–7).

**Fig. 4:**
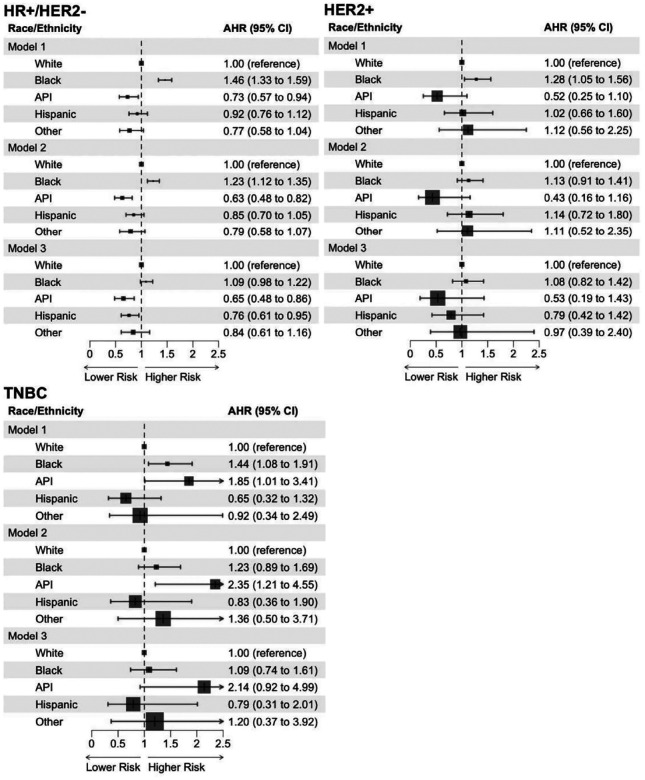
Racial/ethnic differences in overall survival of male patients with breast cancer, stratified by molecular subtype: Cox proportional hazards regression. Abbreviations: API, Asian or Pacific Islander; AHR, adjusted hazard ratio; Cl, confidence interval; HR, hormone receptor; HER2, human epidermal growth factor receptor 2; TNBC, triple-negative breast cancer. **Model 1** included age at diagnosis only. **Model 2** included age at diagnosis, histologic type, AJCC stage group, tumor grade, and Charlson-Deyo comorbidity score. **Model 3** included age at diagnosis, histologic type, AJCC stage group, tumor grade, Charlson-Deyo comorbidity score, percent no high school degree quartile, median household income quartile, type of health insurance, rural-urban area, and facility type.

When stratifying by molecular subtype, Black patients with HR-positive/HER2-negative mBC had a higher risk of mortality than White patients (AHR 1.23, 95% CI: 1.12–1.35, *P* < 0.001) (model 2) (Fig. 4); however, the difference between the two racial groups was no longer significant when further adjusting for socioeconomic characteristics (AHR 1.09, 95% CI: 0.98–1.22, *P* = 0.111) (model 3). Patients with a median household income of $50,354-$63,332 (AHR 0.86, 95% CI: 0.76–0.98, *P* = 0.019) or ≥$63,333 (AHR 0.73, 95% CI: 0.64–0.84, *P* < 0.001) had a lower mortality risk than those of <$40,227 (Supplementary Table 8). Compared to private insurance, no insurance (AHR 1.68, 95% CI: 1.29–2.19, *P* < 0.001), Medicare (AHR 1.21, 95% CI: 1.10–1.33, *P* < 0.001), or Medicaid (AHR 1.73, 95% CI: 1.43–2.09, *P* < 0.001) was associated with an increased mortality risk (Supplementary Table 8). For HER2-positive tumors, the OS rate was similar by race/ethnicity (Fig. 4). Patients with a comorbidity score of ≥ 2 or without insurance experienced worse OS (Supplementary Table 9). Socioeconomic factors were not associated with OS in male TNBC (Supplementary Table 10). In the TNBC cohort, API patients were at an increased mortality risk than White patients (AHR 2.35, 95% CI: 1.21–4.55, *P* = 0.011), after adjusting for clinicopathologic characteristics (Fig. 4).

## Discussion

We compared the mortality of mBC in a large US cohort by racial/ethnic groups, socioeconomic factors across tumor stages and molecular subtypes. Black patients had a higher mortality risk than White patients when adjusting for clinicopathologic factors. However, when further adjusting for socioeconomic indicators, the mortality risk between Black patients and White patients did not vary to a level of statistical significance. Overall, API and Hispanic patients had better OS than White patients. In the TNBC cohort, API patients fared worse OS than White patients, meriting further investigation. Lower median household income quartiles, Medicaid, Medicare, or no insurance, and greater comorbidity scores were associated with a higher mortality risk in mBC.

In this study, Black mBC patients consistently had higher mortality than White patients across tumor stages and molecular subtypes, after adjusting for clinicopathologic characteristics. This racial disparity mirrors trends found in female breast cancer studies.^14^ Notably, however, after further adjusting for socioeconomic indicators, the survival difference between Black patients and White patients was no longer statistically significant. A recent comparative analysis of mBC has also documented a similar OS rate between the two racial groups after controlling for both clinical and sociodemographic factors.^18^ Our findings suggest that alleviating socioeconomic inequities may reduce disparities in mBC mortality between Black patients and White patients.

We also found that after adjusting for clinicopathologic characteristics or further for socioeconomic factors, API or Hispanic patients with mBC displayed non-statistically significant trends toward slightly lower mortality risk compared with White patients, and this was consistent across tumor stages and molecular subtypes. However, it is worth noting that in the TNBC cohort, API patients experienced the lowest 5-year and 10-year OS rates compared with other racial/ethnic categories. This is a surprising result, as previous research in TNBC have described Black men having significantly lower OS rates than White men, without much focus on other racial/ethnic groups displaying the same trend.^19^ This result may be due to the small sample size for the API patient group. It is also possible that these survival disparities may be masked by aggregation of Asian and other Pacific Islander as a racial category.^20^ Between 2010 and 2014, Asian women with TNBC had better survival of any racial demographic with stage I-III tumors but poorer survival in the metastatic setting.^21^ Furthermore, API women experienced the steepest increase of all racial/ethnic groups in breast cancer rates from 2012 to 2021,^14^ combined with a significant increase in TNBC incidence between 2010 and 2019 among those ages ≥ 55 years.^22^ This shift in TNBC incidence and lower survival rates in API women may contain parallels in male TNBC, which warrants future research.

Socioeconomic indicators correlated with mortality disparities in mBC, both overall and when stratified by tumor stage or molecular subtype. Across all mBC patients and within stage I-II and HR-positive/HER2-negative disease, higher median household income quartiles were associated with greater survival rates. An analysis of the 2004–2016 NCDB reported that higher income levels were associated with improved survival in mBC. However, this and other studies have not fully stratified patients based on molecular subtype and/or tumor stage.^11,12,16,18^ Having private health insurance was associated with a lower mortality risk compared to having no health insurance coverage, Medicaid, or Medicare. Patients with a higher median household income had better survival outcomes, aligning with findings from prior studies.^6,18,23^ However, educational attainment, facility type, and rural-urban residence were not significantly or consistently associated with OS across tumor stages and molecular subtypes, though congruent with reports in mBC literature.^11,23^ Collectively, these findings suggest that differences in health insurance and household income may contribute to disparities in mBC mortality.

Several limitations of this study should be acknowledged. First, although the NCDB includes extensive clinicopathologic and certain relevant sociodemographic data, its mortality statistics only include all-cause mortality. It is worth examining disease-specific, progression-related, or other survival outcomes of mBC in future studies. Moreover, there are many unmeasured potential confounders not being collected by the NCDB, such as environmental factors, lifestyle data, personal and family history, and genetic predispositions, that likely influence the survival differences across racial/ethnic groups observed in the current study. Therefore, investigators should consider these key variables in their future analyses. Lastly, given the nature of the NCDB registry and this retrospective study design, the generalizability of our findings may be limited. However, the racial demographics of mBC patients in the NCDB generally reflect the US population, and our findings are consistent with prior studies using the Surveillance, Epidemiology, and End Results data.^12,16^

In conclusion, Black patients with mBC had a greater mortality risk than White patients when controlling for clinicopathologic features; however, the risk was similar between the two racial groups after further controlling for socioeconomic indicators. Compared with White patients, API or Hispanic patients had better survival outcomes, except for API patients who had higher mortality from TNBC. Lower median household income, lack of health insurance coverage or public insurance, and greater comorbidity scores were associated with a higher risk of mortality. Our findings highlight racial/ethnic disparities and socioeconomic determinants of survival outcomes in mBC across tumor stages and molecular subtypes. Strategies to address socioeconomic inequities that impact access to comprehensive cancer care programs and services may help reduce racial/ethnic disparities and improve survival outcomes of the US mBC population.

## Methods

### Study design and data source

This was a retrospective study of real-world data from mBC patients registered in the 2010–2021 National Cancer Database (NCDB), collected by the Commission on Cancer (CoC) of the American College of Surgeons and the American Cancer Society.^24^ The NCDB captures approximately 72% of new cancer diagnoses each year from more than 1,500 CoC-accredited cancer centers in the US.^25,26^ Because NCDB de-identified data does not identify clinical facilities, providers, or patients, the University of Chicago Institutional Review Board exempted the study from review. In compliance with the NCDB’s Data Use Agreement, we suppressed reporting of cell counts < 10 to protect patients’ confidentiality. The study followed the Strengthening the Reporting of Observational Studies in Epidemiology (STROBE) reporting guideline.^27^

### Eligibility and sample selection

Sample selection of male patients diagnosed with invasive breast carcinoma was illustrated in Supplementary Fig. 1. Briefly, patients were eligible if they 1) were at least 18 years of age at diagnosis; 2) were assigned male sex assigned at birth; 3) had tumors classified as stage I, II, III, or IV by the American Joint Committee on Cancer (AJCC) staging system; 4) were diagnosed between 2010 and 2021; and 5) included data on tumor molecular subtype.

#### Measures

Tumor stage was defined by the AJCC staging system and classified as stage I, II, III, or IV.^28^ Molecular subtypes of breast tumors were categorized as HR-positive/HER2-negative, HR-positive/HER2-positive, HR-negative/HER2-positive, and TNBC. Due to small sample sizes, the HR-positive/HER2-positive and HR-negative/HER2-positive groups were combined into a HER-positive (or HER2-enriched) category for regression analyses.

The main outcome of interest was OS, which was defined as the time from the initial breast cancer diagnosis to death from any cause or last patient contact. According to the NCDB, vital status was not available for patients diagnosed in 2021 because of limited time for follow-up. Thus, these patients were not included in our survival analysis. Median follow-up (in months) for the patient cohort was reported.

Racial/ethnic groups comprised non-Hispanic Asian or Pacific Islander (API), non-Hispanic Black, Hispanic, non-Hispanic White, and Other. Other is a racial/ethnic group listed in the NCDB and represents patients who were classified as Other by local cancer registries. The NCDB does not specifically define race/ethnicity classified into Other. Demographic and clinicopathologic characteristics included age at diagnosis, year of diagnosis, percent no high school degree quartile based on residential geographic area (≥ 17.6%, 10.9–17.5%, 6.3–10.8%, and < 6.3%), median household income quartile (<$40,227, $40,227-$50,353, $50,354-$63,332, and ≥$63,333), type of health insurance (Medicaid, Medicare, other government, private, uninsured), rural-urban area, type of facility/cancer program, Charlson-Deyo comorbidity score (0, 1, and ≥ 2), tumor histologic type (ductal, ductal and lobular, lobular, or other), and tumor grade (1 - low, 2 - intermediate, and 3 - high).

### Statistical analysis

First, we described the patient cohort using standard summary statistics. We used analyses of variance or Kruskal-Wallis tests to compare the distributions of continuous variables. Categorical variables were compared using Pearson’s chi-squared tests. For survival analysis, the Kaplan-Meier (K-M) method was used to calculate the median survival time (in months) and estimate 3-year, 5-year, and 10-year OS rates across racial/ethnic groups, overall and stratified by tumor stage and by molecular subtype. We conducted stratified log-rank tests to determine statistical significance when comparing survival functions across racial/ethnic groups, by tumor stage and by molecular subtype. Cox proportional hazards regression models were fit to further assess racial/ethnic and socioeconomic disparities in OS. A stepwise regression approach was employed. Specifically, model 1 included age at diagnosis and race/ethnicity; in addition, model 2 included histologic type, AJCC stage, tumor grade, and Charlson-Deyo comorbidity score; and, in addition, model 3 included percent no high school degree quartile, median household income quartile, type of insurance, rural-urban area, and facility type. A similar approach was implemented in the stratified Cox regression when stratifying by tumor stage and by molecular subtype. Adjusted hazard ratios (AHR) and 95% confidence intervals (95% CI) were calculated. All statistical tests were two-sided at the 0.05 level of significance. All statistical analyses were performed using Stata version 18 (StataCorp, College Station, TX). Forest plots were created using the *forestploter* package in R (R Foundation for Statistical Computing).

## Figures and Tables

**Table 1:** Overall sociodemographic and clinicopathologic characteristics of male patients with breast cancer

	Overall (N = 20470)
**Characteristic**	n (%)
**Age at diagnosis (years)**, mean (SD)	66.2 (12.6)
**Race/ethnicity**	
White	16015 (78.2)
Black	2817 (13.8)
API	503 (2.5)
Hispanic	818 (4.0)
Other	317 (1.6)
**Percent no high school degree quartiles** ^a^	
≥17.6%	3093 (17.5)
10.9–17.5%	4390 (24.8)
6.3–10.8%	5108 (28.9)
<6.3%	5099 (28.8)
**Median household income quartiles** ^b^	
<$40,227	2853 (16.2)
$40,227–$50,353	3601 (20.4)
$50,354–$63,332	4110 (23.3)
≥$63,333	7089 (40.2)
**Type of health insurance**	
Uninsured	407 (2.0)
Private/managed care	7637 (37.3)
Medicaid	1011 (4.9)
Medicare	10702 (52.3)
Other government/unknown	713 (3.5)
**Rural-urban area**	
Metro	17335 (86.8)
Urban	2360 (11.8)
Rural	271 (1.4)
**Facility type/cancer program**	
Community	1775 (8.9)
Comprehensive community	8171 (40.9)
Academic/research	5770 (28.9)
Integrated network	4256 (21.3)
**Charlson-Deyo comorbidity score**	
0	15346 (75.0)
1	3364 (16.4)
≥2	1760 (8.6)
**Histologic type**	
Ductal	17717 (86.6)
Lobular	552 (2.7)
Ductal and lobular	387 (1.9)
Other	1814 (8.9)
**AJCC stage group**	
I	9411 (46.0)
II	6564 (32.1)
III	2725 (13.3)
IV	1770 (8.6)
**Molecular subtype**	
HR+/HER2-	15972 (83.6)
HR+/HER2+	2154 (11.3)
HR-/HER2+	231 (1.2)
TNBC	758 (4.0)
**Tumor grade**	
1	2653 (14.4)
2	9965 (54.1)
3	5796 (31.5)
**Median follow-up time in months** (IQR)	52.8 (28.7, 84.6)

Abbreviations: SD, standard deviation; IQR, interquartile range; API, Asian or Pacific Islander; AJCC, American Joint Committee on Cancer; HR, hormone receptor; HER2, human epidermal growth factor receptor 2; TNBC, triple-negative breast cancer; BCS, breast-conserving surgery.

a Defined as education attainment for patient residence areas and measured by matching the zip code of the patient recorded at the time of diagnosis against files derived from the 2016 American Community Survey data.

b Based on the 2016 American Community Survey data, spanning years 2012–2016 and adjusted for 2016 inflation.

**Table 2:** Distributions of sociodemographic and clinicopathologic characteristics of male patients with breast cancer by race/ethnicity

	White	Black	API	Hispanic	Other	*P* value^a^
**Characteristic**	n (%)	n (%)	n (%)	n (%)	n (%)	
**Age at diagnosis (years)**, mean (SD)	67.2 (12.3)	63.2 (12.6)	63.1 (13.4)	61.0 (13.9)	63.4 (13.3)	< 0.001
**Percent no high school degree quartiles** ^b^						
≥17.6%	1744 (12.6)	841 (34.9)	97 (22.0)	357 (49.6)	54 (18.8)	< 0.001
10.9–17.5%	3272 (23.7)	824 (34.2)	75 (17.0)	150 (20.8)	69 (24.0)	
6.3–10.8%	4294 (31.0)	504 (20.9)	109 (24.8)	140 (19.4)	61 (21.2)	
<6.3%	4522 (32.7)	241 (10.0)	159 (36.1)	73 (10.1)	104 (36.1)	
**Median household income quartiles** ^c^						
<$40,227	1642 (11.9)	944 (39.2)	40 (9.1)	191 (26.6)	36 (12.5)	< 0.001
$40,227–$50,353	2836 (20.6)	490 (20.4)	53 (12.0)	163 (22.7)	59 (20.5)	
$50,354–$63,332	3354 (24.3)	450 (18.7)	70 (15.9)	181 (25.2)	55 (19.1)	
≥$63,333	5967 (43.2)	523 (21.7)	277 (63.0)	184 (25.6)	138 (47.9)	
**Type of health insurance**						
Uninsured	228 (14)	95 (3.4)	13 (2.6)	62 (7.6)	9 (2.8)	< 0.001
Private/managed care	5925 (37.0)	1008 (35.8)	238 (47.3)	328 (40.1)	138 (43.5)	
Medicaid	542 (3.4)	298 (10.6)	55 (10.9)	97 (11.9)	19 (6.0)	
Medicare	8797 (54.9)	1293 (45.9)	189 (37.6)	291 (35.6)	132 (41.6)	
Other government/unknown	523 (3.3)	123 (4.4)	<10 (< 2.0)	40 (4.9)	19 (6.0)	
**Rural-urban area**						
Metro	13267 (85.1)	2557 (92.3)	471 (96.3)	773 (95.8)	267 (88.4)	< 0.001
Urban	2092 (13.4)	189 (6.8)	17 (3.5)	30 (3.7)	32 (10.6)	
Rural	239 (15)	24 (0.9)	<10 (< 1.0)	<10 (<1.0)	<10 (< 1.0)	
**Facility type/cancer program**						
Community	1483 (9.4)	168 (6.2)	40 (8.4)	59 (7.8)	25 (8.2)	< 0.001
Comprehensive community	6660 (42.4)	962 (35.5)	180 (37.7)	269 (35.4)	100 (32.7)	
Academic/research	4176 (26.6)	1032 (38.1)	174 (36.4)	280 (36.8)	108 (35.3)	
Integrated network	3397 (21.6)	550 (20.3)	84 (17.6)	152 (20.0)	73 (23.9)	
**Charlson-Deyo comorbidity score**						
0	12103 (75.6)	1966 (69.8)	396 (78.7)	628 (76.8)	253 (79.8)	< 0.001
1	2601 (16.2)	517 (18.4)	69 (13.7)	132 (16.1)	45 (14.2)	
≥2	1311 (8.2)	334 (11.9)	38 (7.6)	58 (7.1)	19 (6.0)	
**Histologic type**						
Ductal	13950 (87.1)	2365 (84.0)	429 (85.3)	706 (86.3)	267 (84.2)	< 0.001
Lobular	464 (2.9)	60 (2.1)	<10(< 15)	12 (1.5)	<10 (< 3.0)	
Ductal and lobular	307 (19)	38 (1.3)	11 (2.2)	19 (2.3)	12 (3.8)	
Other	1294 (8.1)	354 (12.6)	56 (11.1)	81 (9.9)	29 (9.1)	
**AJCC stage group**						
I	7588 (47.4)	1087 (38.6)	244 (48.5)	357 (43.6)	135 (42.6)	< 0.001
II	5120 (32.0)	930 (33.0)	150 (29.8)	256 (31.3)	108 (34.1)	
III	2052 (12.8)	447 (15.9)	60 (11.9)	124 (15.2)	42 (13.2)	
IV	1255 (7.8)	353 (12.5)	49 (9.7)	81 (9.9)	32 (10.1)	
**Molecular subtype**						
HR+/HER2-	12660 (84.4)	2055 (79.1)	391 (83.2)	618 (82.5)	248 (84.6)	< 0.001
HR+/HER2+	1660 (11.1)	346 (13.3)	48 (10.2)	77 (10.3)	23 (7.8)	
HR-/HER2+	165 (11)	38 (1.5)	13 (2.8)	<10 (< 1.5)	<10 (< 2.5)	
TNBC	521 (3.5)	158 (6.1)	18 (3.8)	45 (6.0)	16 (5.5)	
**Tumor grade**						
1	2161 (14.9)	304 (12.4)	70 (16.0)	93 (12.8)	25 (8.8)	< 0.001
2	7865 (54.2)	1299 (52.8)	237 (54.1)	392 (54.0)	172 (60.6)	
3	4481 (30.9)	856 (34.8)	131 (29.9)	241 (33.2)	87 (30.6)	
**Median follow-up time in months** (IQR)	53.7 (29.3, 85.8)	47.4 (26.2, 78.1)	54.8 (30.0, 83.7)	52.5 (26.7, 80.1)	52.3 (27.3, 85.2)	< 0.001

Abbreviations: SD, standard deviation; IQR, interquartile range; API, Asian or Pacific Islander; AJCC, American Joint Committee on Cancer; HR, hormone receptor; HER2, human epidermal growth factor receptor 2; TNBC, triple-negative breast cancer; BCS, breast-conserving surgery.

a *P* values were calculated using ANOVA or Kruskal-Wallis tests for continuous data and Pearson’s *X*^2^ tests for categorical data.

b Defined as education attainment for patient residence areas and measured by matching the zip code of the patient recorded at the time of diagnosis against files derived from the 2016 American Community Survey data.

c Based on the 2016 American Community Survey data, spanning years 2012–2016 and adjusted for 2016 inflation.

## Data Availability

Data for this study were obtained from the US National Cancer Database. Investigators affiliated with Commission on Cancer-accredited cancer programs can request the National Cancer Database Participant User File by submitting an application to the American College of Surgeons via https://www.facs.org/quality-programs/cancer-programs/national-cancer-database.

## Abbreviations

API: Asian or Pacific Islander
HR: hormone receptor
HER2: human epidermal growth factor receptor 2
TNBC: triple-negative breast cancer
